# Management of open olecranon fractures using clamp-cum-compressor device

**DOI:** 10.4103/0019-5413.45324

**Published:** 2009

**Authors:** Zile Singh Kundu, P Kamboj, SS Sangwan, RC Siwach, Raj Singh, P Walecha

**Affiliations:** Department of Orthopaedics, Physical Medicine, Paraplegia and Rehabilitation, Pt. B. D. Sharma Post Graduate Institute of Medical Sciences, Rohtak - 124 001, Haryana, India

**Keywords:** Clamp-cum-compressor, olecranon fractures, open fractures

## Abstract

**Background:**

Open fractures of olecranon are not a rare occurrence in patients with road traffic accidents particularly motor bike riders who don't use elbow guards. Definitive treatment has to be delayed in many till the wound heals. The present study was conducted to evaluate the results of open fractures of olecranon using clamp-cum-compressor device.

**Materials and Methods:**

Seventeen patients between the ages of 20 and 45 years of open olecranon fractures reported 5-20 days after injury were treated using an indigenous clamp-cum-compressor. All fractures were Mayo type II-A, i.e., displaced, stable and noncomminuted. Four patients had Gustilo-Anderson grade I and 13 had Gustilo-Anderson grade II open fractures. The patients with transverse or short oblique fractures were included in the study. The apparatus was applied under regional anesthesia after thorough washing and debridement of wounds with few loose sutures applied wherever needed. The wounds healed within 2-4 weeks and fractures united within 8-10 weeks. The elbow was mobilized with apparatus still in place. The results were evaluated by MayoElbow performance score.

**Results:**

We achieved excellent results in twelve patients, good in four and poor in one patient, who reported late, hooks of the apparatus were cut through the proximal fragment, leading to union of fracture in elongation and restricted elbow movements.

**Conclusion:**

The apparatus was found to be quite useful in transverse and short oblique fractures with contamination or infection, where internal fixation has to be delayed or avoided.

## INTRODUCTION

Olecranon is fractured quite frequently in adults and open fracture occurs in 2–31% of cases.^1^ Fractures of olecranon mainly result in response to three types of injuries: (a) direct violence such as fall on the point of elbow or a direct blow on the olecranon; (b) indirect violence such as a fall with outstretched hand with elbow in flexion accompanied by a strong contraction of triceps; and (c) thirdly, there may be the combination of the first two. These fractures are either undisplaced or displaced types. When the gap is less 2 mm and does not increase with 90° flexion of the elbow and patient is able to actively extend the elbow against gravity, the fracture is undisplaced type and is managed conservatively.^2^ The displaced fractures are managed by open reduction and internal fixation, tension band wiring for transverse fractures; and plate fixation in comminuted and oblique fractures.^3^,^4^ However, in open fractures, internal fixation has to be delayed till wound heals, otherwise infection occurs leading to osteomyelitis and/or septic arthritis. This will ultimately cause stiff elbow and post-traumatic secondary osteoarthritis. In view of these problems, a clamp-cum-compressor was designed to achieve fracture union as well as wound healing in open fractures simultaneously. For open fractures, the described modality of treatment is half ring external fixator, which is technically more demanding as compared to our innovative device. We report a series of seventeen patients with open olecranon fracture to evaluate the results of clamp-cum-compressor device.

## MATERIALS AND METHODS

Seventeen male patients of open olecranon fractures, with age 20–45 years presented in the Orthopaedics department constitute the study. Right side was involved in 11 patients. All fractures were Mayo type II-A, i.e., displaced, stable and noncomminuted. Four patients had Gustilo-Anderson grade I and 13 had Gustilo-Anderson grade II open fractures. The mode of injury in 15 patients was fall on out-stretched hand and direct trauma in the remaining two patients. Eight patients reported on fifth day, six on seventh day, two on tenth day and one patient reported after twenty days of injury. Two patients had ipsilateral fractures of clavicle and one had fracture of fifth metacarpal, which were managed conservatively. The patients with only short oblique or transverse isolated open fractures without comminution were included in the study. The patients with associated intra-articular fractures of distal humerus and elbow subluxation/dislocation were not included in the study. The standard antero-posterior and lateral X-rays of the elbow were taken; wound swabs were sent for culture sensitivity. The wounds were washed with four to five liters of normal saline and tetanus prophylaxis was given to all patients. The apparatus was applied under regional anesthesia on same day of reporting.

### Apparatus

**Figure 1:**
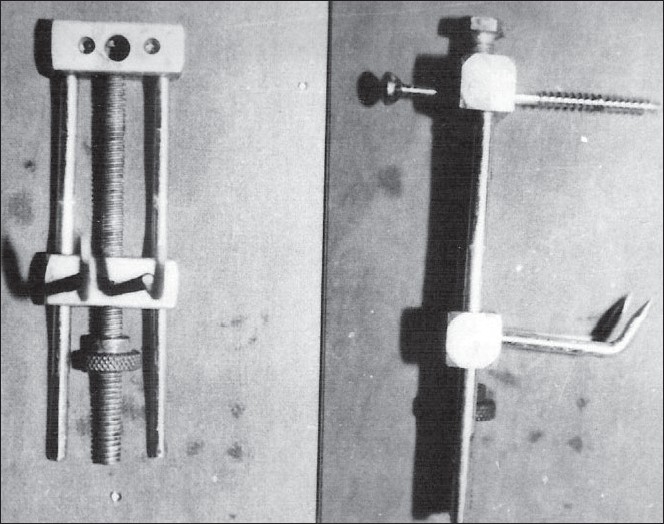
Clamp-cum – compressor apparatus

The apparatus was designed and fabricated from 316 L stainless steel [Figure 1]. It consists of two metallic bars. One of the bars is static on which two thin smooth side rods and one central threaded rod are attached. This bar has a central hole for passage of a malleolar screw, which is passed through this hole and stabilized with a side-bolt. The second metallic bar is dynamic and can glide over the two side rods and central threaded rod with the help of a bolt. On this bar, two hooks/prongs are attached for anchoring the fractured proximal fragment. These hooks are made from 3-mm Steinmann's pins. One screw and two hooks make a tripod configuration for stability. The bolt over the central threaded rod is used for achieving apposition and compression at fracture site by the movement of dynamic metallic bar along with two hooks.

### Operative procedure

All operations were performed under brachial or axillary block and image intensifier control.

**Figure 2:**
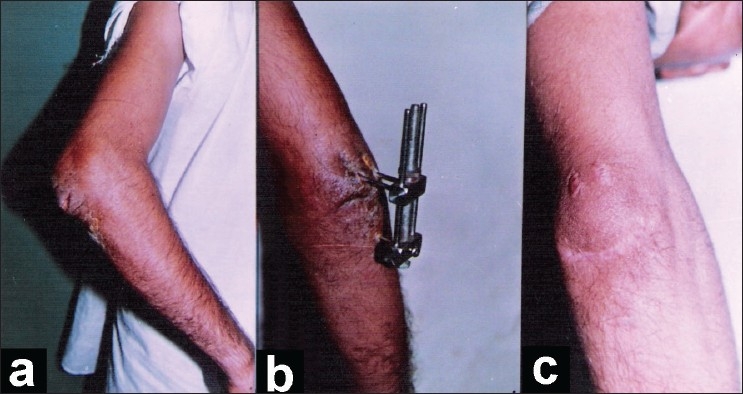
Clinical photograph showing transverse infected wound at fracture site (a), after application of apparatus (b) and after wound and fracture healing (c)

In lateral position arm hanging by the chest, The wounds were thoroughly scrubbed and debrided. A stab incision was made on dorsal aspect of ulna about 2 cm distal to the articular part of olecranon; a malleolar screw of 50-60 mm length was passed through the hole in the distal fixed bar of the apparatus and then to the ulna. The direction of tip of the screw should be in the opposite cortex just distal to coronoid process to attain good fixation and avoid entry into the joint. Then two stab incisions were made on both sides of triceps tendon near its insertion on the olecranon. The two hooks of the apparatus were introduced through these stabs and anchored to the fractured proximal fragment of the olecranon. The hook should not be too far medially to avoid injury to the nerve. The elbow was extended to achieve reduction and the hooked fragment was compressed using the central compression bolt by moving the dynamic bar to achieve apposition and compression. The malleolar screw was stabilized in the hole with the help of a side-bolt [Figure 2].

The transverse wounds of skin remained well apposed without sutures. In 4 cases, loose sutures were applied to leave space for drainage. The apposition and reduction of fracture was confirmed post-operatively with antero-posterior and lateral X-rays of the elbow. Anatomical reduction was aimed and achieved in all the cases.

The patients were encouraged immediate post-operative active elbow movements to avoid stiffness. Broad spectrum intravenous antibiotics, including one of the third generation cephalosporins (cefotaxime) combined with one aminoglycoside (gentamicin) were started till the report of culture sensitivity was obtained and the antibiotics were changed accordingly. After one week, they were switched to oral antibiotics according to culture sensitivity for another 2 weeks.

**Figure 3:**
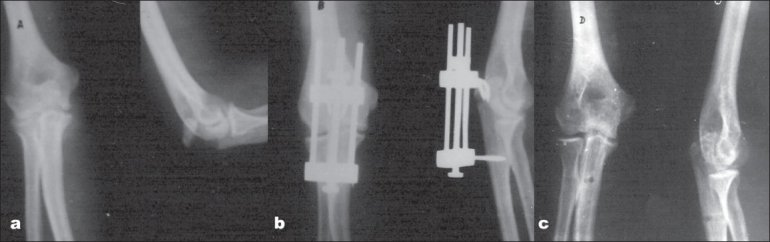
X-rays of the elbow (anteroposterior and lateral views) showing (a) fracture olecranon (b) after clamp application (c) during follow-up after union of fracture and removal of apparatus

Patients were advised daily wound washing and dressing with povidone iodine lotion and also cleansing of hooks and screw sites. Emphasis was laid on active flexion and extension of the elbow to avoid stiffness. They were followed up weekly for the first three weeks and usually by that time wounds had healed. X-rays were taken at three, six and nine weeks intervals after surgery. Then, the patients were followed up at three months interval in the first year and six monthly in the subsequent year. X-rays were taken at each follow-up. The apparatus was removed after clinical as well as radiological union, i.e., after eight to ten weeks of operation when the bridging callus was appreciable [Figure 3]. The hooks and screw sites were thoroughly washed with saline povidone iodine solution, which healed uneventfully in all cases. The patients were advised physiotherapy including active elbow flexion and extension exercises along with pronation and supination of forearm.

## RESULTS

The follow-up ranged from 2 to 4 years. The results were evaluated using Mayo elbow performance score.^5^ The ulnar nerve was intact in all the cases pre as well as post-operatively. Patients were discharged from the hospital 3 days after surgery, after confirming that hooks were well anchored in proximal fragment and fracture site was adequately compressed and apposed, thus achieving anatomical reduction.

In 13 cases, *Staphylococcus aureus* was the infecting organism. There was mixed bacterial infection in four cases. Most of the bacteria were sensitive to amoxicillin and clavulinic acid combination and the third generation cephalosporins. Wound healing took 2–4 weeks and fractures united in 8–10 weeks. Secondary plastic procedure like skin grafting or flap coverage was required in none of the cases. The apparatus was removed after 8 weeks in 10 patients, 9 weeks in 5 and 10 weeks in 2 cases post-operation.

The results were evaluated according to the Mayo Elbow Performance Score [Table 1] for pain, range of motion, stability and functional outcome.^5^ Excellent results were achieved in 12 patients, good in four and poor in one. Initially, there was mild stiffness of the elbow in all patients till the apparatus was in place due to pain at the site of hooks and screws. But after removal of the apparatus followed by physiotherapy, almost full range of movements were achieved within three months.

**Table 1:** Mayo Elbow performance score

Pain (max. 45 points)
None (45 points)
Mild (30 points)
Moderate (15 points)
Severe (0 points)
Mean
Range of motion (max. 20 points)
Arc > 100 degrees (20 points)
Arc 50 to 100 degrees (15 points)
Arc < 50 degrees (5 points)
Mean
Stability (max. 10 points)
Stable (10 points)
Moderately unstable (5 points)
Grossly unstable (0 points)
Mean
Function (max. 25 points)
Able to comb hair (5 points)
Able to feed oneself (5 points)
Able to perform personal hygiene tasks (5 points)
Able to on shirt (5 points)
Able to put on shoes (5 points)
Mean
Mean total (max. 100 points)
Score greater than 90: Excellent
Score 75–89: Good
Score 60–74: Fair
Score below 60: Poor

**Figure 4:**
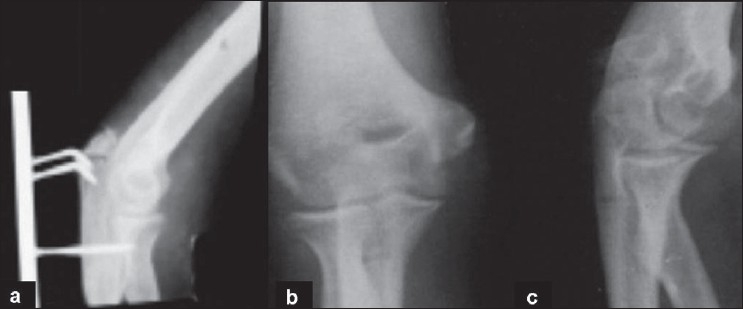
(a) X-ray of the elbow (lateral view) showing implant cut out. X-ray (anteroposterior (b) and lateral views (c)) of the same patient showing union of fracture in elongation.

One patient who reported after 20 days of injury with infection had poor outcome. He had triceps contracture and fractured fragment was slightly osteoporosed; so the hooks cut through the proximal fragment during flexion movement and the fracture malunited in elongation [Figure 4]. He also had stiffness of the elbow and pain.

## DISCUSSION

Closed displaced fractures of olecranon are managed by open reduction and internal fixation. The standard treatment includes figure of eight tension band wiring, using two parallel K-wires or a partially threaded cancellous screw for noncomminuted, stable transverse fractures; and plate osteosynthesis for comminuted and oblique fractures.^3^,^4^,^7^–^10^ However, in open contaminated fracture, internal fixation will not only introduce the infection deeper inside, leading on to the complications such as osteomyelitis and septic arthritis, but·also infection will persist till the implant is inside, thereby causing stiffness. Further, in infected cases, the internal fixation may become loose, leading to failure of fixation. The hardware becomes symptomatic, which is most common complication of internal fixation and needs removal in majority of the cases.^11^–^13^

Nonoperative treatment has its own disadvantages such as (i) failure to reduce the fracture leads to its union in elongation resulting in limitation of power of extension; (ii) articular incongruity leads to post-traumatic arthritis; (iii) displaced olecranon will block the full extension of elbow; (iv) longer immobilization in full extension leads to failure to regain flexion of the elbow.^2^ If one waits till the wound heals, there will be fibrosis during healing process and triceps will be shortened and subsequent surgery will require more dissection, more soft tissue and periosteal stripping, thereby causing delayed union and more chances of stiffness.

Application of simple external fixator in open olecranon fractures using Schanz pins is not possible due to relatively small proximal fragment, which will not accommodate at least two Schanz pins for stability.^14^ Moreover, if such a frame is tried, it may cause splintering and comminution of the proximal fragment. Ilizarov circular external fixator has also been tried and used for open fracture of olecranon;^15^ however, passage of wires in a small proximal fragment is quite difficult; the assembly is cumbersome and costly.

With the intention to avoid above mentioned problems, clamp-cum-compressor device has been used for treatment of open olecranon fracture in this study. This will not only allow the wound care but anatomical reduction and early fracture healing also. The apparatus works on the basic “tension-band principle.” The tension band is placed external to the skin surface and compression is achieved with the help of central compression bolt through the two hooks and malleolar screw anchored to the bone fragments, and there will be further compression and apposition with flexion of elbow.

The apparatus allows good access to wound for cleansing as well as secondary plastic procedure if needed. The patients are more comfortable being devoid of bulky plaster cast and are able to do active elbow movements, thus avoiding stiffness. Another advantage is that the compression at the fracture site can be achieved any time during follow up with central turnbuckle if there is loosening or gap at fracture site. Implant removal is an outdoor procedure. So unlike internal fixation, a second relatively major procedure of removal of hardware is not required. The hooks and screw are applied away from the wound and fracture site; so there is less chance of osteomyelitis at fracture site. The fractured fragment is well attached to the soft tissues and the biology is well preserved as no incision, dissection or periosteal stripping is needed, thus achieving early healing of fracture. It can be easily applied even in small proximal fragment without causing splintering and comminution; hence, there is no need of excision of the fragment.

The transverse wounds usually need no sutures and in other wounds only 2–3 loose sutures are applied just to cover the exposed bone and leaving the space for drainage. In the present series, none of the patient needed skin grafting or flap coverage. By the time wound healed and infection settled, there was simultaneous progression of fracture healing also.

The apparatus has been found to be well suited for Mayo type II-A, i.e., displaced, stable and noncomminuted fractures; and upto grade II open fractures only. It is not suitable for comminuted fractures as there are chances of hooks cutting through. In our series wounds healed in 2-4 weeks with apparatus in situ providing sufficient space to daily dress the wounds and simultaneously elbow mobilization was started. The fracture also united in 8-10 weeks. The only one case had proximal cut through of clamp which later healed by malunion giving poor results.

The main shortcoming of the apparatus is that it is successful in transverse and short oblique fractures only.

## CONCLUSION

It is concluded that this method of management will be quite useful for the patients with infected and contaminated open fractures of olecranon. There is no need to wait for the wound healing as after the application of the apparatus, both wound healing and fracture union progress simultaneously. The apparatus is quite economical and is very simple to construct and apply.
